# Multi-omics characterization and therapeutic liability of ferroptosis in melanoma

**DOI:** 10.1038/s41392-022-01067-y

**Published:** 2022-08-10

**Authors:** Yi He, Yu Dong, Yong Chen, Guanxiong Zhang, Hailun Zhang, Guang Lei, Yanhua Du, Xiang Chen, Youqiong Ye, Hong Liu

**Affiliations:** 1 Department of Dermatology, Hunan Key Laboratory of Skin Cancer and Psoriasis, Hunan Engineering Research Center of Skin Health and Disease, Xiangya Clinical Research Center for Cancer Immunotherapy, Xiangya Hospital, Central South University, Changsha, China; 2 Shanghai Institute of Immunology, Department of Immunology and Microbiology, Shanghai Jiao Tong University School of Medicine, Shanghai, China; 3 Department of Musculoskeletal Surgery, Fudan University Shanghai Cancer Center, Shanghai, PR China; 4 Department of Oncology, Shanghai Medical College, Fudan University, Shanghai, PR China; 5 Department of Research and Development, Beijing GAP Biotechnology Co., Ltd, Beijing, China; 6 Department of Radiation Oncology, Hunan Cancer Hospital and The Affiliated Cancer Hospital of Xiangya School of Medicine, Central South University, Changsha, China

**Keywords:** Biological sciences, Genome informatics, Cancer metabolism, Skin cancer

**Dear Editor**,

Ferroptosis, an iron-dependent form of programmed cell death driven by excessive lipid peroxidation,^1^ has emerged as a promising and effective strategy for triggering cancer cell death.^2,3^ Insufficient understanding of the role of ferroptosis in melanoma progression and the tumor microenvironment (TME) has limited the development of ferroptosis-targeted therapy and combinations with other therapeutic strategies.

Here, we constructed a ferroptosis score (FPS) model to quantify the ferroptosis status of melanoma patients based on 32 ferroptosis-related genes (FRGs) screened from the FerrDB^4^ database that were associated with patient outcomes and could accurately differentiate patient status (Supplementary Figs. 1, 2; see Methods). The univariate Cox hazard analysis demonstrated that FPS was the most significant OS protective factor compared with 50 cancer hallmark gene sets (Fig. 1a), and patients with high FPS had better overall survival (Supplementary Fig. 2g). Multivariate Cox regression analysis demonstrated that FPS is an independent predictor by considering the confounding factors (e.g., age, gender, and tumor stage; Supplementary Fig. 2h). The reliability of the FPS as a prognostic factor was validated in four independent melanoma cohorts with 416 patients (Supplementary Fig. 3).

**Fig. 1:**
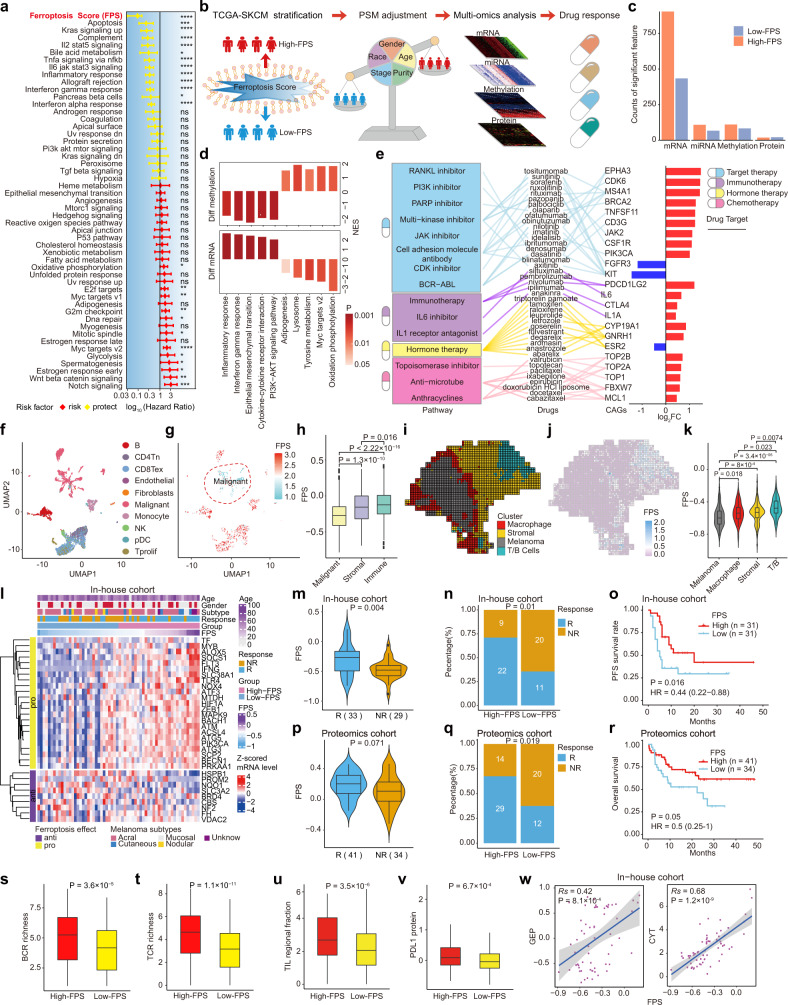
Comprehensive analyses of ferroptosis: implications for cancer therapeutic liability. **a** Univariate Cox hazard analysis demonstrated that Ferroptosis Score (FPS) was the top significant protect factor for overall survival compare with all the 50 hallmarks of cancer in the TCGA-SKCM cohort. **b** The overview of integrated analysis of multi-omics using propensity score algorithm (PSM) and drug response. **c** The number of each significant altered molecular feature identified by PSM in the high-FPS group and the low-FPS group from TCGA-SKCM samples. **d** Enrichment of KEGG and Hallmark gene sets calculated by GSEA for mRNA expression (left panel) and DNA methylation (right panel). The color indicates the statistically significant enriched gene set (adjust P-value < 0.05). **e** Ferroptosis-associated CAGs targeted by FDA-approved drugs. The right bar graph displays the significant alterations (log_2_FC) CAGs in the high-FPS group vs. low-FPS group. **f** UMAP embeddings of single-cell RNA-seq profiles from GSE115978. **g** UMAP plot show FPS expression profiles of whole tissue cells from GSE115978. **h** Boxplot show the difference of FPS in malignant cells, stromal cells, and immune cells. **i** Spatial enhanced-resolution clustering performed by the BayesSpace algorithm identified four clusters corresponding to the original histopathological annotations. **j** Spatial heatmap shows the difference of FPS expression among four clusters at the enhanced-resolution condition. **k** Comparison analysis between the four clusters highlighted spatial differences in the enrichment of our FPS model. **l** Clinical features of in-house patient cohort treated with anti-PD1 and the relative expression level of pro-FRGs and anti-FRGs. Each column represents an individual patient. **m**, **p** The difference in the FPS between R (responders) and NR (non-responders) patients of in-house cohort (**m**; transcriptome level) and proteomics cohort (p; protein level). **n**, **q** The proportion of patients with different responses to immunotherapy of in-house cohort **n** and proteomics cohort **q**. **o**, **r** Kaplan-Meier curves show progression free survival (o; in-house cohort) and overall survival (r; proteomics cohort) in the high-FPS (red) and low-FPS (blue). **s**–**v** The expression of BCR richness **s**, TCR richness **t**, TIL regional fraction **u**, and PDL1 protein level **v** between high-FPS and low-FPS group from TCGA-SKCM cohort. **w** The Spearman correlation between FPS and ICB response-related features, including GEP and CYT. Two-sided Wilcoxon test was used in **h**, **k**, **m**, **p**, and **s**–**v**, chi-square test was used in **n** and **q**, two-sided log-rank test was used in **o** and **r**. BCR, B cell receptor; TCR, T cell receptor; CAGs, clinical actionable genes; GEP, T cell-inflamed gene expression profile; CYT, cytolytic activity; TIL,Tumor infiltration lymphocyte

To identify the molecular features related to ferroptosis in melanoma, we applied a propensity score matching (PSM) algorithm to appropriately control clinical confounders (e.g., tumor purity, stage) and calculated the molecular difference between high-FPS and low-FPS groups (Fig. 1b, Supplementary Fig. 1e; see Methods). We identified ferroptosis-associated features in four molecular levels, including 1338 mRNAs, 205 significant miRNAs, 193 methylation sites, and 49 proteins (Fig. 1c; Supplementary Table 5), suggesting that ferroptosis in melanoma may affect multiple molecular levels. Then we performed GSEA analysis between high and low FPS group in mRNA and methylation levels, respectively. The results showed immune-related pathways (*e.g*., inflammatory response, interferon gamma response) were enriched in patients with high-FPS, while those related to oxidation phosphorylation and *MYC* targets v2 were enriched in patients with low-FPS (Fig. 1d, bottom panel; Supplementary Table 6). Ferroptosis associated methylation features showed opposite direction of function enrichment comparing to ferroptosis associated mRNA features (Fig. 1d, up panel; Supplementary Table 6), suggesting ferroptosis-associated mRNA expression patterns were partially due to alterations in DNA methylation level. Next, we observed that a high FPS was strongly correlated with sensitivity to ROS inducers (PD-DI; Piperlongumine; Supplementary Fig. 5) and ferroptosis inducers (Erastin; RSL; ML162; ML210; Supplementary Fig. 5), suggesting the robustness of the FPS model for defining ferroptosis status. Furthermore, we found multiple ferroptosis associated clinically actionable genes targeted by ﻿Food and Drug Administration (FDA)-approved drugs were altered at the mRNA level (Fig. 1e). For example, *PDCD1LG2* (*PD1* ligand) was highly expressed in patients with high-FPS (Fig. 1e), suggesting that *PD1* inhibitors, such as nivolumab and pembrolizumab, could have better efficacy in high-FPS tumors.

Ferroptosis was found to be associated with the tumor immune response according to multi-omics analysis (Fig. 1d, Supplementary Fig. 4). We further explored ferroptosis status alterations at a single-cell resolution to better characterize intra-tumor heterogeneity. Employing graph-based principal component clustering combined with marker-based annotations, we classified cells from GSE115978 into 10 clusters (Fig. 1f; Supplementary Fig. 6a). We found that the FPS was generally lowest in malignant cells, followed by fibroblasts, and the highest FPS was observed in immune cells (Fig. 1g,h) and confirmed this in another independent melanoma scRNA-seq dataset (GSE72056; Supplementary Fig. 6b–e). To investigate the regulation of ferroptosis in the TME at two-dimensional spatial level, we classified a spatial transcriptome melanoma dataset from a previous study^5^ into macrophage, stromal, melanoma, and T/B cell enrichment regions (Fig. 1i; Supplementary Fig. 7) and found the heterogeneity of the FPS in these four regions. The FPS was higher in the border region with high infiltration of macrophages than in the tumor center and the FPS was higher in the region with T/B cell enrichment than in the stromal regions (Fig. 1j,k). These suggest the heterogeneity of ferroptosis status among different cell populations at single-cell and spatial levels. In particular, there was a predominant difference in the FPS between malignant cells and immune cells, thus inducing ferroptosis in tumor cells may be a potential treatment strategy.

To investigate the power of the FPS to predict ICB therapy efficacy, we collected our in-house cohort of 62 melanoma patients with anti-PD-1 treatment, including 33 responders and 29 non-responders (Supplementary Table 8; see Methods). Consistently, anti-FRGs tended to be highly expressed in non-responders, while pro-FRGs were more likely to be highly expressed in responders (Fig. 1l), and the responders showed significantly higher FPS that non-responders (Wilcoxon test, *P* = 0.004; Fig. 1m). Among these 62 patients, 22 out of 31 patients (71%) with high FPS were responders to anti-PD-1 treatment, which is significantly higher than the percentage of patients with low FPS (22/31 versus 11/31; chi-square test: *P* = 0.01; Fig. 1n). In addition, patients with a higher FPS had longer progression free survival (PFS) (log-rank test, *P* = 0.016; Fig. 1o). We validated FPS associated with immunotherapy benefit in four independent melanoma bulk RNA-seq cohorts, including TCGA-SKCM, PRJEB23709, phs000452.v3, and GSE91061 (Supplementary Fig. 8). Further, in the scRNA-seq melanoma cohort (GSE120575) treated with anti-PD-1 therapy, we found patients with a higher FPS in both pre-treatment and post-treatment benefited from immunotherapy (Supplementary Fig. 9). In addition, we found FPS associated with immune response in protein level. The protein levels of anti-FRGs and pro-FRGs showed distinguishing expression patterns in responders and non-responders in anti-PD-1 treated cohort with proteomics (Supplementary Fig. 10a, b). Multi-Cox analysis revealed that protein-based FPS can be independent prediction model to predict the immunotherapy efficacy (Supplementary Fig. 10c) and associated with benefit of anti-PD-1 therapy (Fig. 1p–r). Taken together, these findings suggest the robustness of the FPS model as a potential biomarker to predict immunotherapy response based on bulk, single-cell transcriptome, and proteomic-level data.

To explore the potential mechanism of ferroptosis in immunotherapy, we performed an association analysis between the FPS and the immune response-related biomarkers. We observed a significantly positive correlation between the FPS and the T cell-inflamed gene expression profile (GEP; Fig. 1w; Supplementary Figs. 10d, 11a) and cytolytic activity (CYT; Fig. 1w; Supplementary Fig. 11b) in 17 independent melanoma cohorts. In addition, B cell receptor (BCR) richness (Fig. 1s), T cell receptor (TCR) richness (Fig. 1t), the tumor-infiltrating lymphocyte (TIL) regional fraction (Fig. 1u), and the PDL1 protein level (Fig. 1v), were significantly higher in the high-FPS group than those in the low-FPS group. These results highlight that ferroptosis may be associated with immunogenicity and that patients with a high FPS may tend to bear “hot tumors”, accompanied by a higher TCR/BCR clone richness, higher CYT score, GEP level, and immune infiltration level and thus an activated immune system; these features may explain, at least in part, the survival advantage and the greater benefit of ICB treatment in the high FPS group.

Our study provides a comprehensive multi-omics understanding of the effects of ferroptosis on prognosis, the TME, and multiple therapies, especially immunotherapy of melanoma, based on bulk, single-cell, spatial transcriptome, and proteomics analyses (Fig. 1). These findings highlight the potential for cancer therapy that induce ferroptosis as single agents or in combination with other targeted therapies and immunotherapies.

## Supplementary information

Supplementary Materials.docx

Supplementary tables.xlsx

## Data Availability

The bulk/single cell RNA sequencing, proteomics data, spatial transcriptome data and clinical information of melanoma patients, or patients treated with immune checkpoint blockade were described in method section “Data collection and processing”. The resources, tools used in our analyses were described in each method section in the methods. We developed an R package to allow users to calculate ferroptosis score, identify multidimensional ferroptosis-associated molecular features, and conduct function enrichment analysis for their own data. The R package and codes have deposited on the Github (https://github.com/Yelab2020/FPSOmics).

## References

[1] Dixon SJ (2012). Ferroptosis: an iron-dependent form of nonapoptotic cell death. Cell.

[2] Lei, G., Mao, C., Yan, Y., Zhuang, L. & Gan, B. Ferroptosis, radiotherapy, and combination therapeutic strategies. *Protein Cell* (2021). 10.1007/s13238-021-00841-y PMC8563889 33891303

[3] Ma S, Henson ES, Chen Y, Gibson SB (2016). Ferroptosis is induced following siramesine and lapatinib treatment of breast cancer cells. Cell Death Dis..

[4] Zhou, N. & Bao, J. FerrDb: a manually curated resource for regulators and markers of ferroptosis and ferroptosis-disease associations. *Database* **2020**, (2020). 10.1093/database/baaa021 PMC7100629 32219413

[5] Thrane K, Eriksson H, Maaskola J, Hansson J, Lundeberg J (2018). Spatially resolved transcriptomics enables dissection of genetic heterogeneity in stage III cutaneous malignant melanoma. Cancer Res..

