# Supplementary material for: Multi-omics characterization and therapeutic liability of ferroptosis in melanoma
Source: Signal Transduct Target Ther. 2022 Aug 10;7:268. doi: 10.1038/s41392-022-01067-y (PMC9363465; doi:10.1038/s41392-022-01067-y)
Supplement: Supplementary file 1 — Supplementary Materials.docx [file 41392_2022_1067_MOESM1_ESM.docx]

**Supplementary Materials for**

**Multi-omics characterization and therapeutic liability of ferroptosis in melanoma**

**Yi He^1#^, Yu Dong^2#^, Yong Chen^3,4#^, Guanxiong Zhang^1^, Hailun Zhang^6^, Guang Lei^5^, Yanhua Du^2^, Xiang Chen^1,*^, Youqiong Ye^2,*^, Hong Liu^1,*^**

#These authors contributed equally;

*e-mail: [chenxiangck@126.com](mailto:chenxiangck@126.com); [youqiong.ye@shsmu.edu.cn](mailto:youqiong.ye@shsmu.edu.cn); [hongliu1014@csu.edu.cn](mailto:hongliu1014@csu.edu.cn)

**This PDF file includes:**

Materials and Methods

Supplementary Fig 1 to 11

Supplementary Reference

**Materials and Methods**

# Data collection and processing

**Clinical Tissue Samples Collection**

Responders or non-responders to anti-PD1 treatment melanoma patients’ paraffin sections were collected from Xiangya Hospital and Fudan University Shanghai Cancer Center. All tissue samples were collected in compliance with the informed consent policy. Clinical information is summarized in Supplementary Table 8 in the Supporting Information.

**RNA sequencing and data processing**

In the in-house cohort, a total of 62 pre-treatment tumor specimens derived from melanoma patients with anti-PD1 treatment were used for RNA sequencing (RNAseq). RNA-Seq reads were adaptor trimmed and the data quality was assessed with the FastQC software (https://github.com/s-andrews/FastQC) before any data filtering criteria was applied. After obtaining the BAM files, reads were mapped onto the human reference genome (GRCh38.p12 assembly) by using HISAT2^1^ software with default parameters. The mapped reads were assembled into transcripts or genes by using Stringtie^2^ software and the genome annotation file (hg38_ucsc.annotated.gtf). To eliminate the bias of sequencing depths and gene lengths, the relative abundance of the transcript/gene was measured by a normalized metrics, TPM (Transcripts per million mapped reads) and log2-transformed. And the normalized expression matrix was available at Supplementary Table 9.

**Melanoma datasets collection**

mRNA expression, miRNA expression, protein expression, methylation expression, and clinical data (*e.g,*, age, gender, stage, smoking, race, overall survival) from skin cutaneous melanoma (SKCM) samples from The Cancer Genome Atlas (TCGA) were downloaded from the TCGA data portal (https://portal.gdc.cancer.gov/)^3,4^. The tumor purity of TCGA-SKCM samples was obtained from TIMER: Tumor Immune Estimation Resource (http://cistrome.org/TIMER/download.html)^5^ and <https://doi.org/10.5281/zenodo.253193>^6^ and integrated. The richness of T cell receptor/B cell receptor (TCR/BCR) among TCGA-SKCM samples were available at [https://gdc.cancer.gov/about-data/publications/panimmune^6^.](https://gdc.cancer.gov/about-data/publications/panimmune.) 12 GEO skin cutaneous melanoma cohorts without treatment from Gene-Expression Omnibus (GEO) (https://www.ncbi.nlm.nih.gov/geo/) were included for further analysis (detail information; see Supplementary Table 4). The data was analyzed using R (version 3.6.0) and R Bioconductor packages.

**Immunotherapy-associated datasets collection**

In addition, multiple datasets with anti-PD-L1/PD1/CTLA4 cohort were collected in the study (Supplementary Table 4) to investigate the association between FPS and immunotherapy efficacy and prognosis. The Riaz cohort^7^ (GSE91061: Anti-PD1-treated advanced melanoma (Nivolumab)), The Jung cohort^8^ (GSE135222: Anti-PD-L1 or anti-PD1-treated lung cancer) were obtained from Gene-Expression Omnibus (GEO) (https://www.ncbi.nlm.nih.gov/geo/); The Gide cohort^9^ (PRJEB23709: anti-PD-1 monotherapy and anti-PD-1/anti-CTLA-4 Combined Therapy) was downloaded from SRA database **(**<https://www.ncbi.nlm.nih.gov/bioproject/)>. The Liu/VanAllen cohort^10,11^ (phs000452.v3: anti-PD1/CTLA4-treated metastatic melanoma), The Miao cohort^11^ (phs001493: anti-PD1 treated renal cell carcinoma) were downloaded from dbGaP database (<https://www.ncbi.nlm.nih.gov/gap/>). Gene expression and clinical information of these datasets with immunotherapy cohort were downloaded. Processed proteomics data and clinical data of melanoma treated with anti-PD1 were obtained from Beck et al^12^.

**Melanoma single cell and spatial transcriptome datasets collection**

Processed gene expression profiles for melanoma were retrieved from TISCH (http://tisch.comp-genomics.org/) under accession numbers GSE72056^13^ (4645 single cells isolated from 19 patients profiling malignant, immune, stromal, and endothelial), GSE115978^14^ (7186 high-quality scRNA-seq profiles including malignant, immune, stromal, and endothelial from 33 human melanoma tumor), and GSE120575^15^ (16291 immune cells from 48 tumor samples of melanoma patients treated with checkpoint inhibitors). The spatial data of melanoma obtained from BayesSpace package^16^.

**Evaluation of ferroptosis-related genes**

We downloaded all the ferroptosis-related genes (FRGs) in the Ferrdb database, which contains 259 genes. The genes in the FerrDB^17^ database have four different confidence levels: “Validated”, “Screened”, “Predicted”, and “Deduced”. Next, we kept the 149 FRGs that belonging to the “Validated” confidence level from the 259 genes, which is the most reliable genes that requires convincing evidence from strict tests such as pharmacological or genetic inhibition or activation test. Then, 140 protein coding FRGs (Supplementary Table 1) were divided into two categories according to their regulatory direction, including ferroptosis-promote genes (pro-FRGs) and ferroptosis-inhibit genes (anti-FRGs). The gene lists were retained, when the pro-FRGs as a protect factor in melanoma prognosis, while anti-FRGs as a risk factor. Finally, we filtered out to 32 core FRGs correlated with TCGA-SKCM prognosis (Supplementary Fig.2a; Supplementary Table 1).

We further explored the expression pattern of these 32 ferroptosis-related genes (FRGs). We calculated pairwise correlations among the expression of the 32 FRGs in melanoma (Supplementary Fig. 2b) and identified two correlation clusters of FRGs: pro-FRGs (marked in red labels) were significantly positively correlated with other pro-FRGs but negatively correlated with anti-FRGs (marked in blue labels), and vice versa. Furthermore, we applied nonnegative matrix factorization (NMF) algorithms^18^ to classify 470 TCGA-SKCM samples into two clusters based on the expression profiles of the 32 FRGs with an optimal k of 2, and 196 melanoma patients were categorized into Cluster 1, while the other 274 melanoma patients were categorized into Cluster 2 (Supplementary Fig. 1c; Supplementary Table 2). In the prognostic analysis of ferroptosis status, patients in Cluster 1 showed a survival advantage compared to those in Cluster 2 (Supplementary Fig. 1d; log rank test, P = 1.2×10^-4^). We found that Cluster 1, with a favorable prognosis, had a significantly higher FPS than Cluster 2 (Supplementary Fig. 1e; Wilcoxon test, P = 1.7×10^-38^).

**Construct the FPS model and estimate the ferroptosis status**

The FPS model to represent the ferroptosis status was establish based on the expression data for genes of core promote ferroptosis components (pro-FRGs) including *ACSL4, ALOX5, NOX4, TF, ATG3, MAPK9, ATG5, BECN1, PRKAA1, SCP2, BACH1, HIF1A, MTDH, PI3KCA, SOCS1, TLR4, ATM, IFNG, ZEB1, FLT3, ATF3, MYB, SLC38A1*; and negative core components (anti-FRGs) of *SLC3A2, CBS, NQO1, PROM2, HSPB1, VDAC2, NF2, FH, BRD4*. We calculated enrichment score (ES) of pro-FRGs and anti-FRGs using single sample gene set enrichment analysis (ssGSEA) in the R package ‘GSVA’^19^, the ferroptosis score (FPS) to computationally dissect the ferroptosis status of the tissue samples, cancer cell line and single cell was defined by the differences of ssGSEA score between the ES of pro-FRGs minus anti-FRGs. The FPS model was validated in cell line data treat with two ROS-induce agents and four classical ferroptosis inducers from CTRP (Fig. 4) (https://portals.broadinstitute.org/ctrp.v2.1/)^20,21^.

FPS = ssGSEA_Score _(pro-FRGs)_ - ssGSEA_Score _(anti-FRGs)_

**Stratification and multi-omics analysis of tumor samples from TCGA-SKCM cohort**

The TCGA-SKCM samples were divided into two parts by the median cut-off of the ferroptosis scores (FPS), and the samples were defined as high-FPS and low-FPS samples, respectively. We further used the MW method of propensity score matching (PSM) algorithm to balance the effects of potential confounders^22^, including age, gender, tumor purity, race, tumor stage, and examined the balance by comparing standardized difference before and after PSM (standardized difference < 0.05). Subsequently, we compared the molecular difference of multi-omics between high ferroptosis score and low ferroptosis score in TCGA-SKCM. In order to decrease random noise in feature identification, permutation test was repeated 100 times via randomly selecting the high-FPS or low-FPS samples. Significant features for four molecular types were identified by the criterion: mRNA expression |fold change| > 2, FDR < 0.05; miRNA expression |fold change| > 2, FDR < 0.05; total protein and DNA methylation: |difference| > 0.1, FDR < 0.05.

**Gene Set Variation Analysis (GSVA) and pathways enrichment analysis**

In order to study the differences of different ferroptosis status in cancer hallmarks pathway, we used "GSVA" R package^19^ to conduct GSVA enrichment analysis. The gene set ‘h.all.v6.1.symbols’, ‘c2.cp.kegg.v6.2’ were retrieved from the MSigDB database (<http://software.broadinstitute.org/gsea/msigdb/index.jsp>)^23^. Pathway Enrichment analysis was performed by using the fgsea package^24^ and the clusterProfiler Package^25^.

**Calculating the GEP/CYT score**

GEP level in each sample was computed based on the GEP gene signature from Ayers et al^26^ by performing gene set variation analysis (GSVA). We obtained the CYT level by calculating the geometric mean of the gene expression of two cytolytic markers (*GZMA* and *PRF1*)^27^.

**Proteomics data analysis of melanoma datasets**

We calculated the ferroptosis score (FPS) based on ferroptosis-associated protein groups, and divided the Beck cohort into two parts according to the median cut-off (high-FPS group vs. low-FPS group). We further used the PSM algorithm to balance the effects of potential confounders, including age at diagnosis, gender, and tumor stage. The fgsea package was used to functionally annotate the differentially expressed proteins. Kaplan-Meier comparative survival analyses between high-FPS and low-FPS were also assessed using log rank test. Statistical analysis was two-side.

**Single cell RNA-seq analysis of melanoma datasets**

We merging all samples of corresponding cohort (GSE115078, GSE72056, and GSE120575) into a single Seurat object and performed data pre-processing, normalization, integration, and clustering using the R package Seurat v3^28^. We obtained and preprocessed the melanoma spatial sample using BayeSpace R package (http://www.bioconductor.org/packages/release/bioc/vignettes/BayesSpace/inst/doc/BayesSpace.html). Principal component analysis (PCA) was then performed on the top 2,000 most highly variable genes (HVGs). Single-cell ferroptosis scores were calculated using the FPS model algorithm described above. Uniform manifold approximation and projection (UMAP) embeddings of single-cell RNA-seq profiles show the annotated cell types and FPS expression.

**Statistical analysis**

Wilcoxon rank sum test was used to compare the differences. Receiver operating characteristic (ROC) curve was performed to verify the predictive power of the model. Univariate Cox regression model was used to calculate the hazard ratio (HR) between cancer hallmarks pathway GSVA score and FPS. Survminer package was used to determine the cutoff point of survival information for each dataset based on the association between FPS and patient overall survival. To find the maximum rank statistic and reduce the calculated batch effect, the "surv-cutpoint" function was used to dichotomy FPS and all potential cutting points were repeatedly tested, then the patient samples were divided into the high-FPS group and the low-FPS group according to the maximum selected log-rank statistics. Kaplan-Meier comparative survival analyses for prognostic analysis were generated, and the log-rank test was used to determine the significance of the differences. To assess whether FPS is an independent predictor, we included age, gender, and stage as variables to perform multivariate Cox regression model analysis and multivariable logistic regression. Spearman and distance correlation were used to calculate the correlation coefficient of 32 FRGs expression. The association between FPS and immune feature and cancer hallmark were also calculated by spearman correlation. (|*Rs*| >= 0.2 and FDR < 0.05 as statistical significantly correlation). All statistical analysis was two-side and considered P < 0.05 as statistical significance.

**Supplementary Figures**


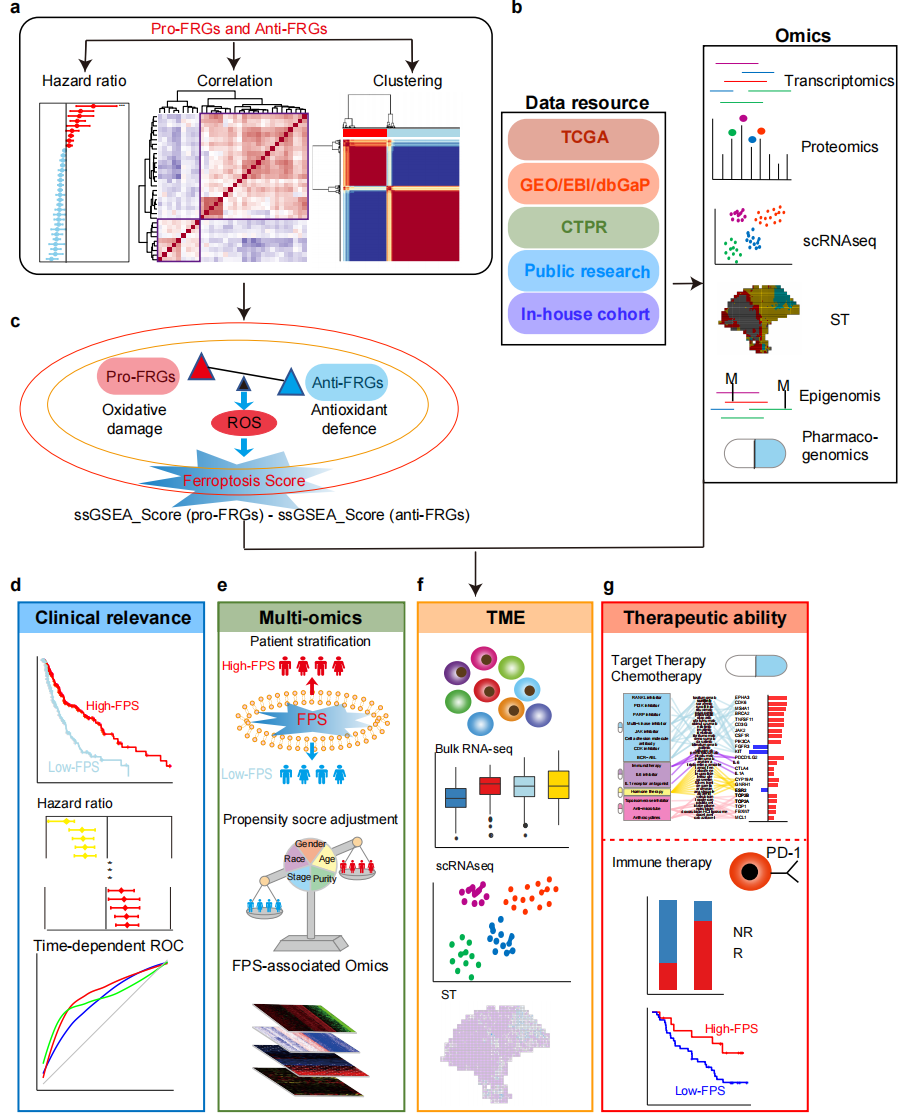


**Supplementary Figure 1. Overview of study design.** Flowchart of the steps in the performed analysis. FRGs, ferroptosis-related genes; FPS, ferroptosis score; PD-1, programmed death 1; TCGA, the Cancer Genomics Atlas; GEO, Gene Expression Omnibus; Lipid ROS, lipid reactive oxygen species; ST, spatial transcriptome; scRNAseq, single cell RNA sequencing; ssGSEA, single sample Gene Set Enrichment Analysis; TME: tumor microenvironment; ROC: receiver operating characteristic.


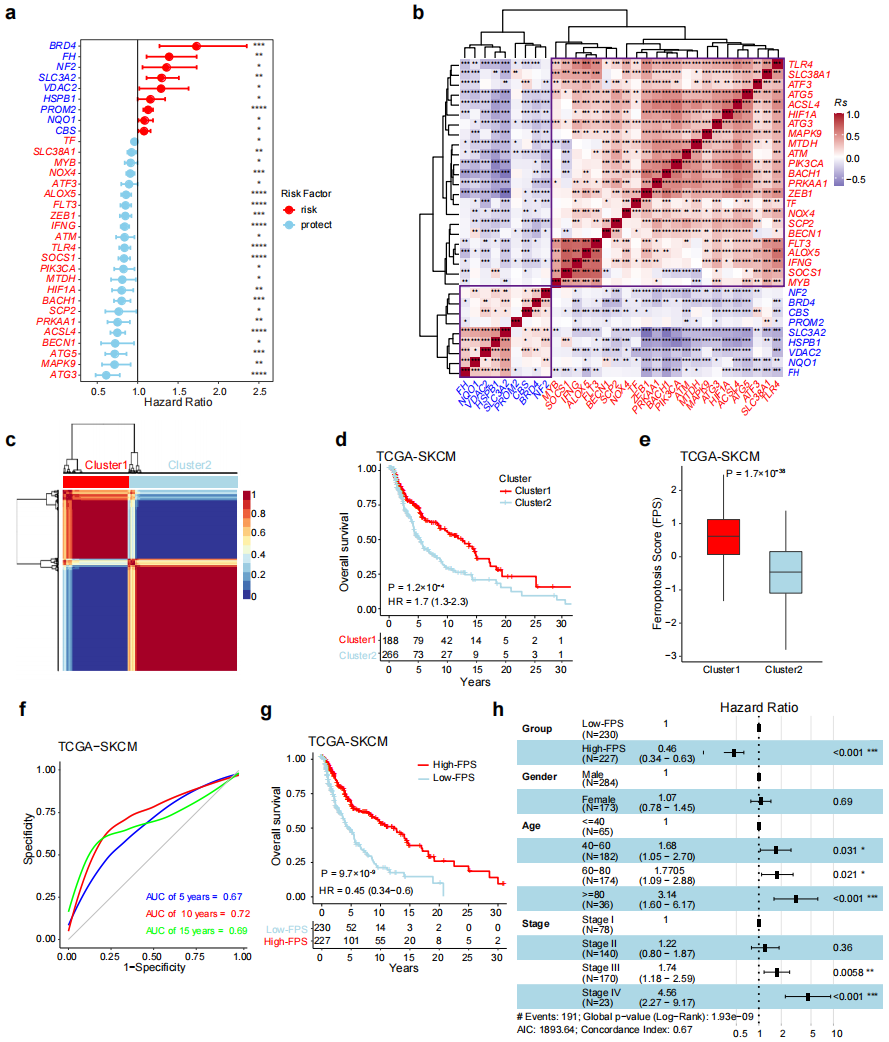


**Supplementary Figure 2. Ferroptosis affect the prognosis of melanoma patients independently of other factors.** (a) Univariate Cox regression analysis of overall survival based on gene expression for 32 Ferroptosis genes in TCGA-SKCM cohort. (b) Heatmap shows a positive (red) and negative (blue) correlation among 32 Ferroptosis-related genes in TCGA-SKCM cohort. *P < 0.05, **P < 0.01, and ***P < 0.001, as determined by the Spearman correlation analysis. Red labels denote pro-FRGs, while blue labels denote anti-FRGs. (c) TCGA-SKCM samples were divided into two clusters using NMF consensus clustering based on 32 Ferroptosis-associated genes. (d) Kaplan-Meier curves compare overall survival between two Ferroptosis clusters, Cluster1 (red) and Cluster2 (light-blue), in TCGA-SKCM. The grouping of TCGA-SCM samples is shown at the bottom of the chart. (e) Differences in the FPS between Cluster1 (red) and Cluster2 (light-blue). (f) The predictive value of FPS in patients among the TCGA-SKCM cohort (AUC: 0.67, 072, and 0.69; 5, 10, and 15-years of overall survival). (g) Kaplan-Meier curves show that patients with higher FPS (red) exhibited better overall survival in the TCGA-SKCM cohorts. (h) The Multivariate Cox regression model analysis, which included the factors of FPS, gender, age, tumor stage. The length of the horizontal line represents the 95% confidence interval (CI) for each group. The vertical dotted line represents the hazard ratio (HR) of all patients shown by the forest plot. Two-sided Wilcoxon test was used in e, two-sided log-rank test was used in d and g.


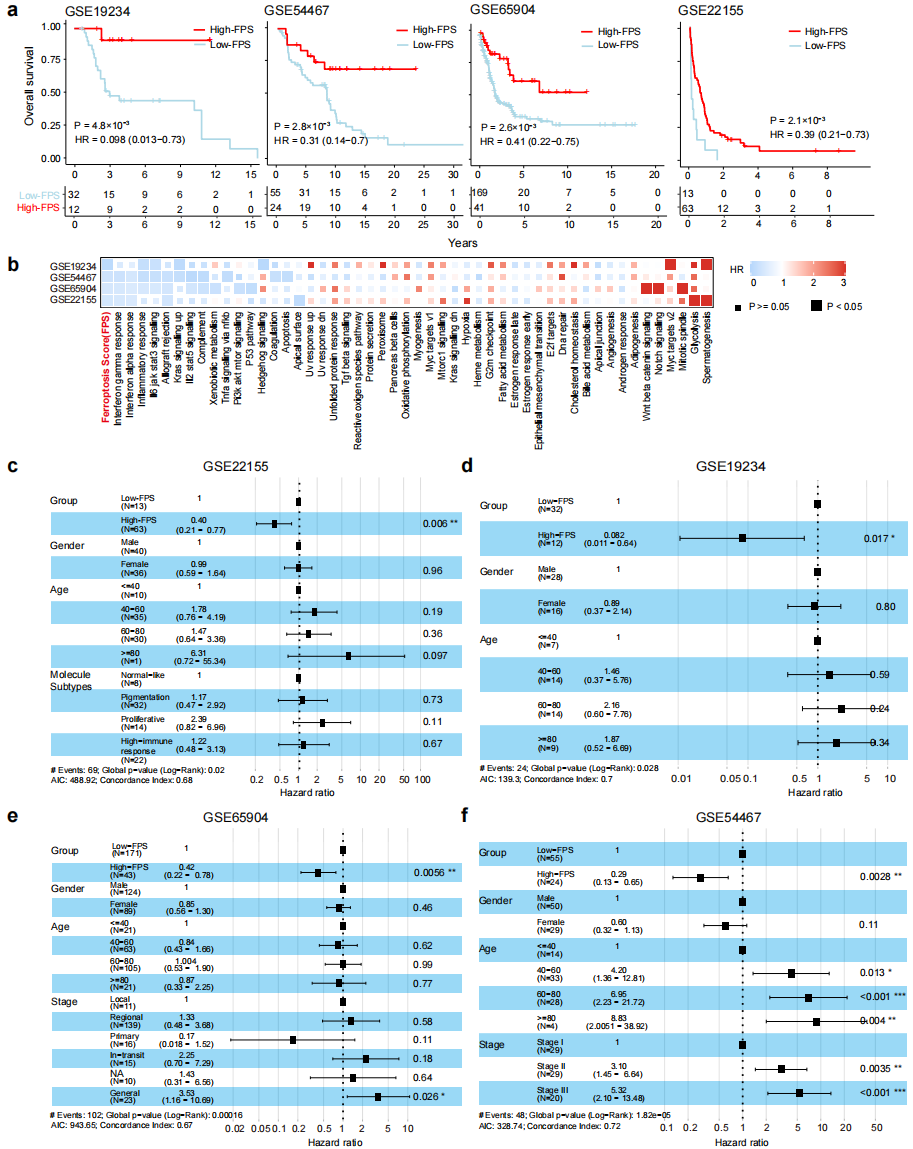


**Supplementary Figure 3. Validation of FPS as a primary prognostic factor in other independent cohorts.** (a) The prognostic value of FPS was validated in four independent cohorts (GSE19234, GSE54467, GSE65904, GSE22155), P<0.05 in the two-sided log-rank test was considered statistically significant. (b) Univariate Cox hazard analysis was performed to evaluate the prognostic capacity of FPS (top) and 50 cancer hallmarks among 4 independent GEO Datasets. (c-f) Forest plot shows FPS independently other clinical features (*e.g.*, gender, age, stage, molecular subtypes) influencing the prognosis of melanoma patients by the multivariate cox regression model analysis across other four melanoma GEO cohorts.


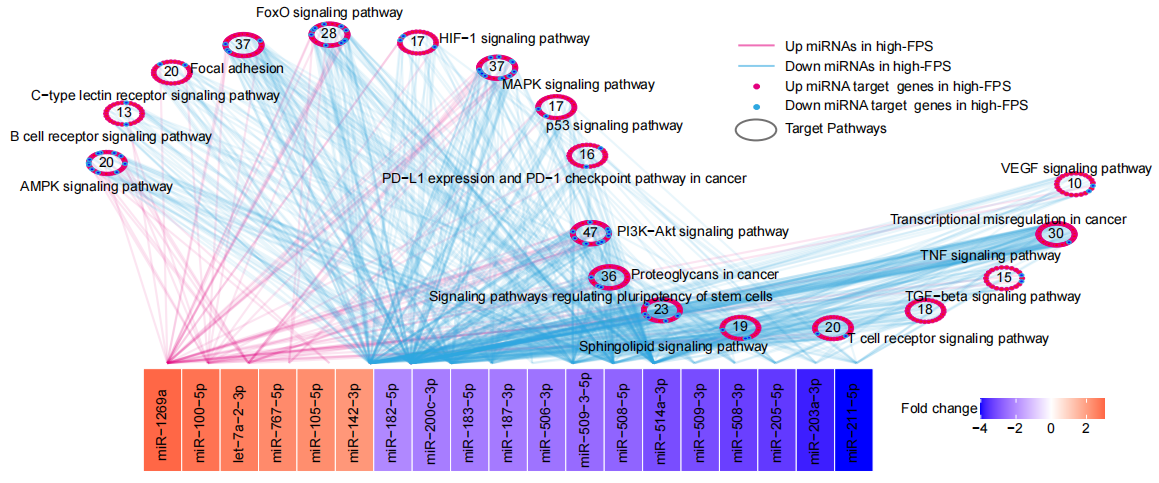


**Supplementary Figure 4**. **Associations between Ferroptosis status and miRNA expression and signaling pathways.** Differences in miRNA-targeted signaling pathways in the TCGA-SKCM cohort between the high-FPS and low-FPS groups. The red line indicates a upregulate of miRNA in the high FPS group, and the blue line indicates a downregulate of miRNA in the high FPS group. Red dots correspond to miRNA-targeted genes highly expressed in the high FPS group, and blue dots correspond to miRNA-targeted genes highly expressed in the high FPS group. The circle represents a signaling pathway enriched with targeted genes. Heatmap shows the differences in miRNA expression levels between high-FPS and low-FPS groups.


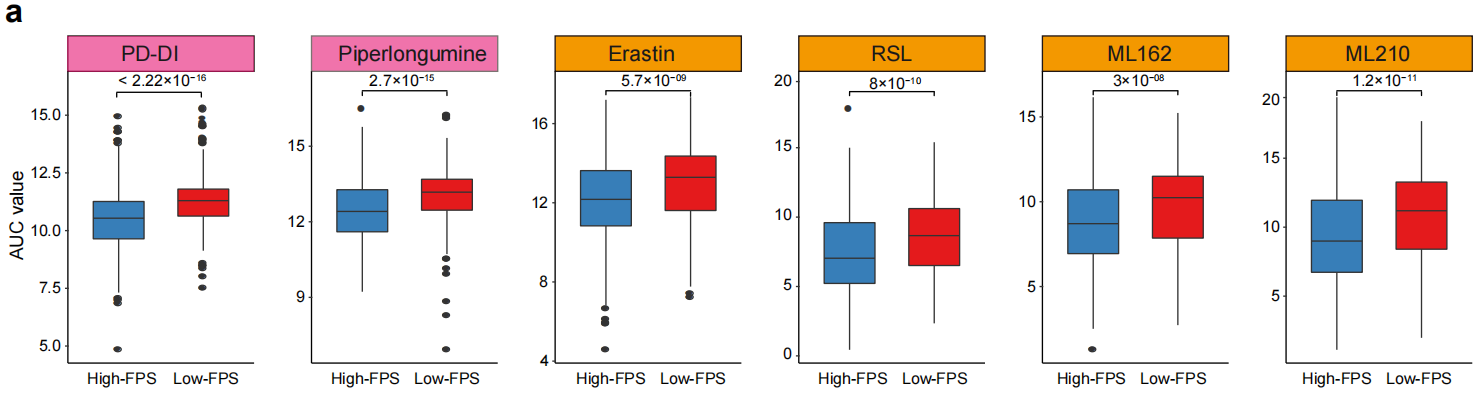


**Supplementary Figure 5. The** **robustness of the FPS model for defining ferroptosis status.** (a) The difference of area under the curve (AUC value) between high-FPS and low-FPS groups across 835 cancer cell lines form CTRP database treated by two natural ROS inducer agents (pink column; PD-DI; Piperlongumine) and four classical ferroptosis inducing agents (marked by yellow column; Erastin; RSL; ML162; ML210). P-value is determined by two-side wilcoxon test.


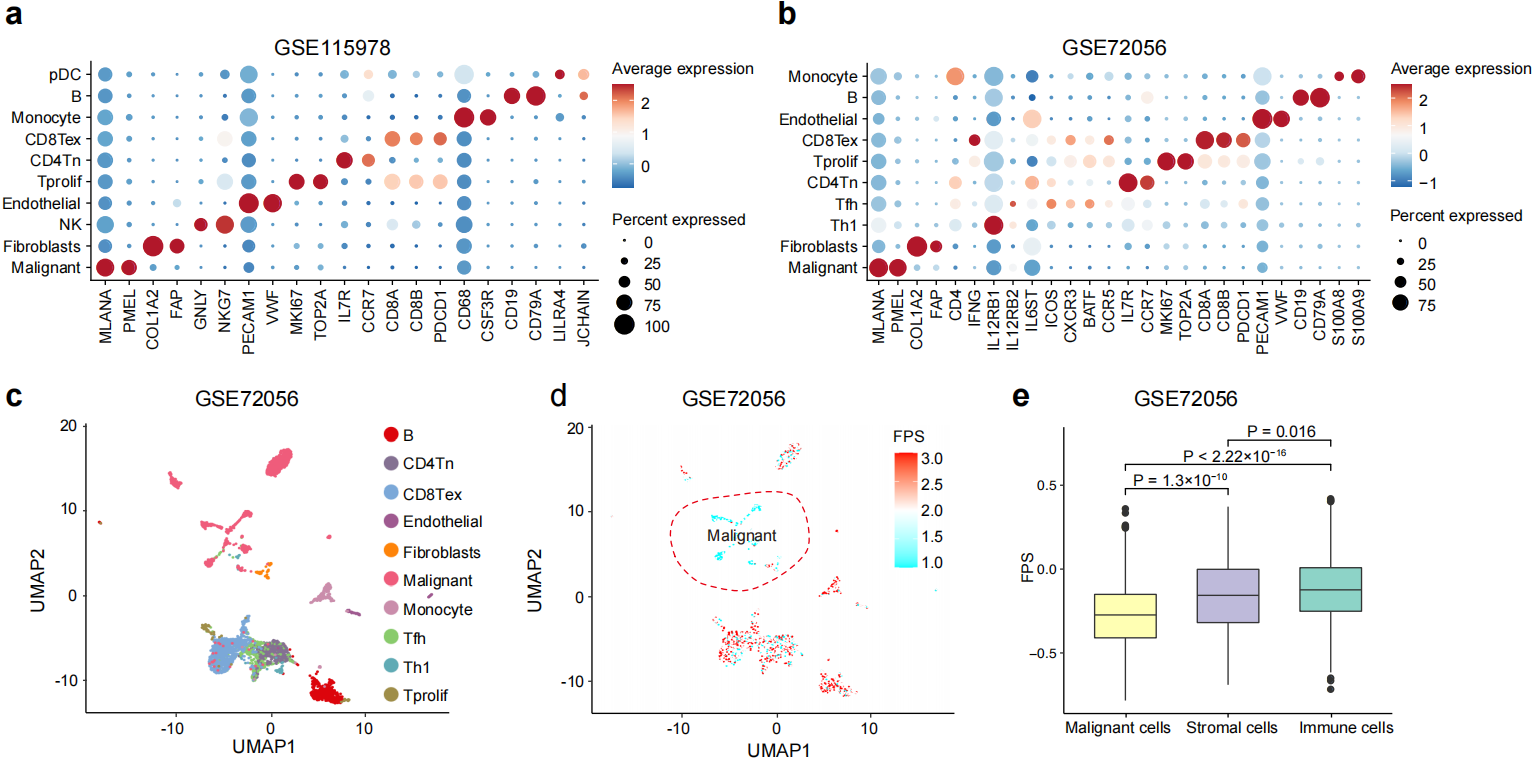


**Supplementary Figure 6. Association between Ferroptosis state and tumor microenvironment at single cell resolution**. (a-b) DotPlot for marker genes of each subtype in single cell transcriptomic profiles, including GSE115978 (a), GSE72056 (b). (c) UMAP embeddings of single-cell RNA-seq profiles from GSE115978 (a) and GSE72056 (b) melanoma cohort. And the cohort show 10, 14 clusters respectively, colored by cell types. (d) UMAP plot show FPS expression profiles of whole tissue cells. (e) Bar plot show the difference of FPS in Malignant cells, stromal cells, and immune cells. P-value is determined by two-sided Wilcoxon test.


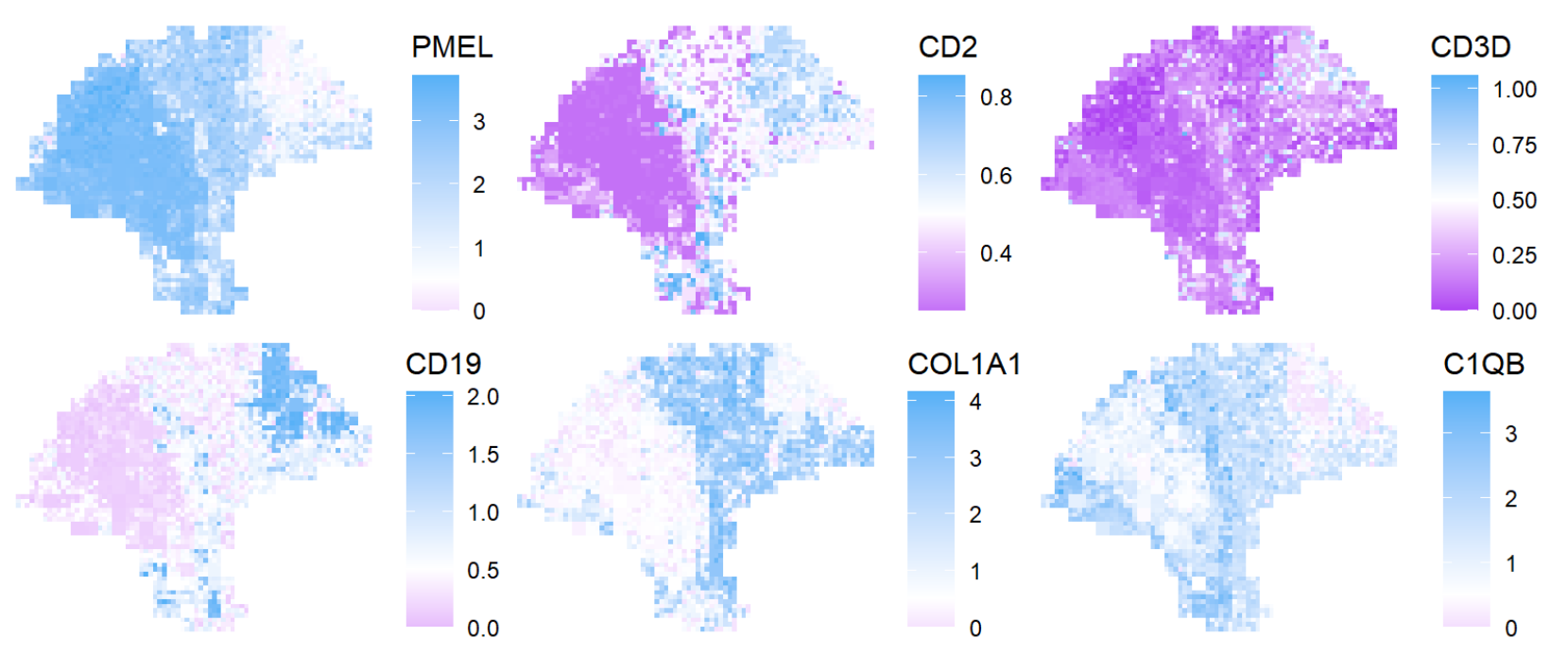


**Supplementary Figure 7.** The expression of cell-types specific markers at spatial transcriptome. Spatial expression plots for marker genes of tumors cell (PMEL), T cells (CD2, CD3D), B cells (CD19), macrophages (C1QB), and fibroblasts (COL1A1).


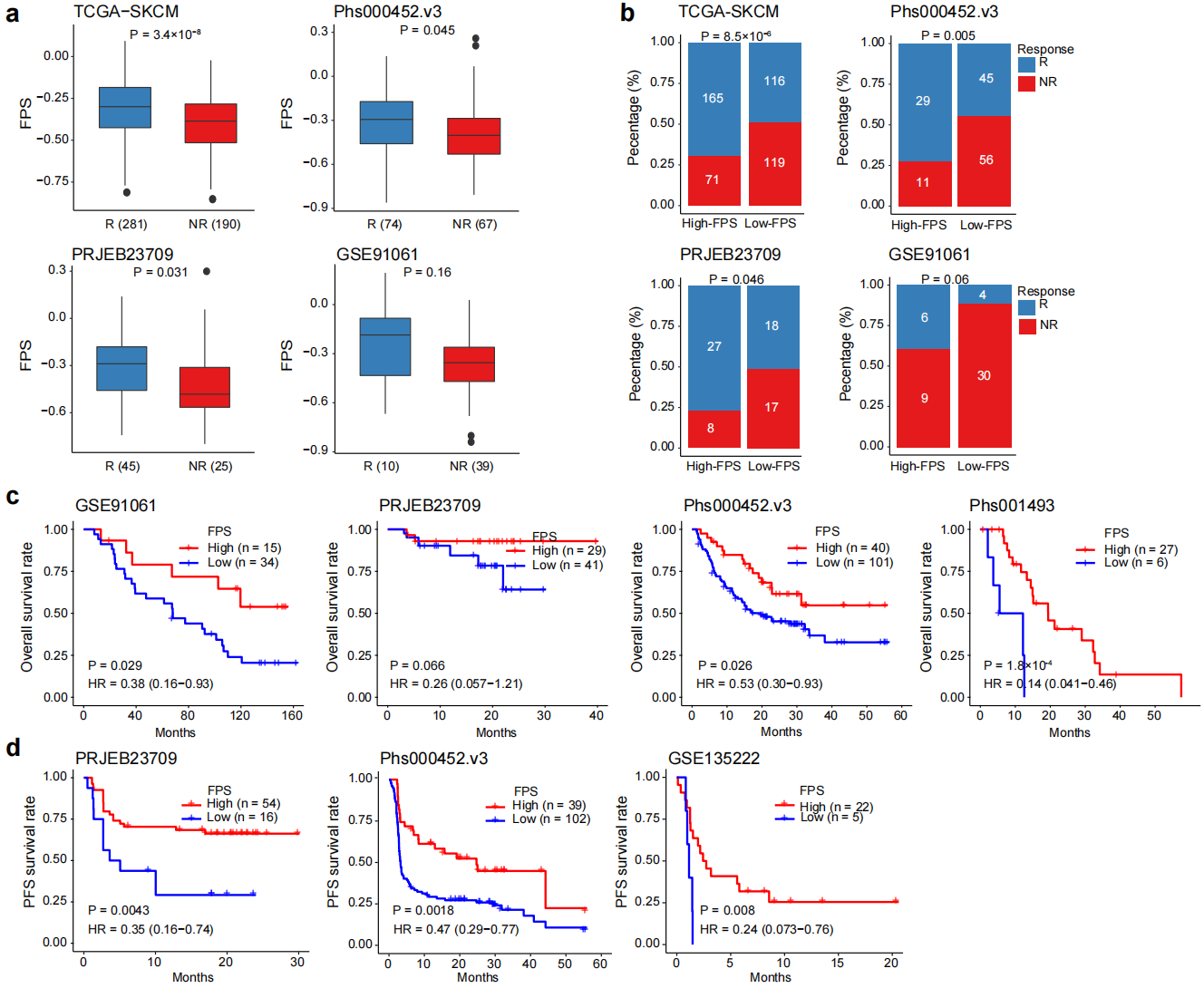
**Supplementary Figure 8. The relationship between FPS and efficacy prediction of immunotherapy in other independent cohorts.** (a) The difference in the FPS between R (responders) and NR (non-responders) patients of TCGA-SKCM cohort with TIDE-predicted ICB response and the three melanoma ICB cohorts. (b) The proportion of patients with different responses to immunotherapy. (c-d). Kaplan-Meier curves show overall survival (c) and Progression-Free Survival (d) in the high-FPS (red) and low-FPS (blue) groups before ICB treatment of in-house cohort, melanoma cohorts (PRJEB23709; phs000452.v3; GSE91061), renal cell carcinoma cohort (phs001493), lung cancer cohort (GSE135222). Two-sided Wilcoxon test was used in a, chi-square test was used in b (TCGA-SKCM; PRJEB23709; phs000452.v3), fisher's exact test was used in b (GSE91061), two-sided log-rank test was used in c and d. TME, Tumor microenvironment; TIDE, Tumor Immune Dysfunction and Exclusion; ICB, Immune checkpoint blockade.


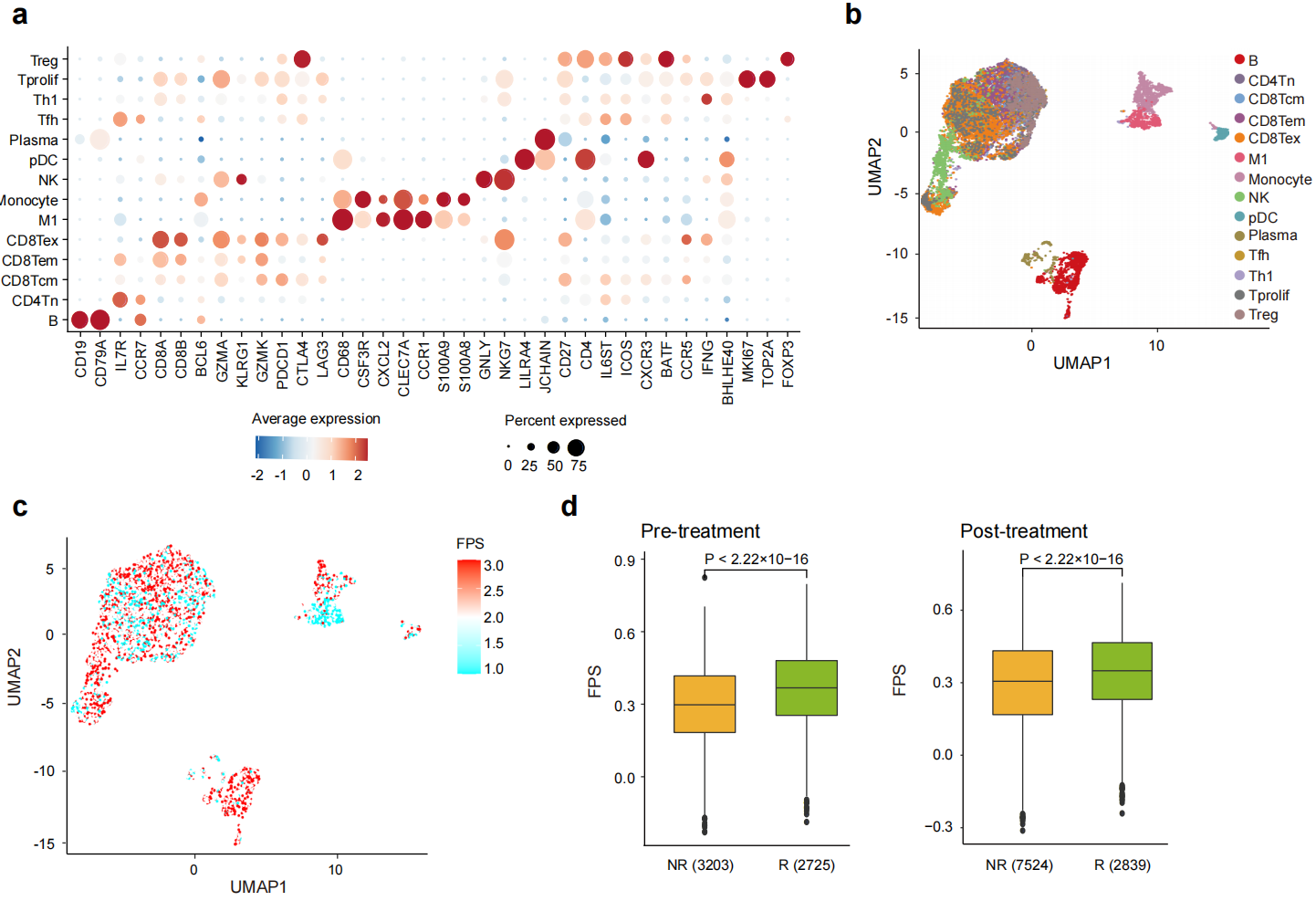


**Supplementary Figure 9. The relationship between FPS and efficacy prediction of immunotherapy at single cell atlas. (a)** DotPlot for marker genes of each subtype in single cell transcriptomic profiles. (b) UMAP embeddings of single-cell RNA-seq profiles from GSE120575 melanoma cohort. And the cohort show 14 clusters, colored by cell types. (c) UMAP plot show FPS expression profiles of whole tissue cells. (d) Bar plot shows the expression difference of FPS among distinct clinical response (responder or non-responder) in pre-treatment and post-treatment conditions of immune checkpoint blockade (GSE120575). The Wilcoxon test was used to determine the statistical significance of the difference.


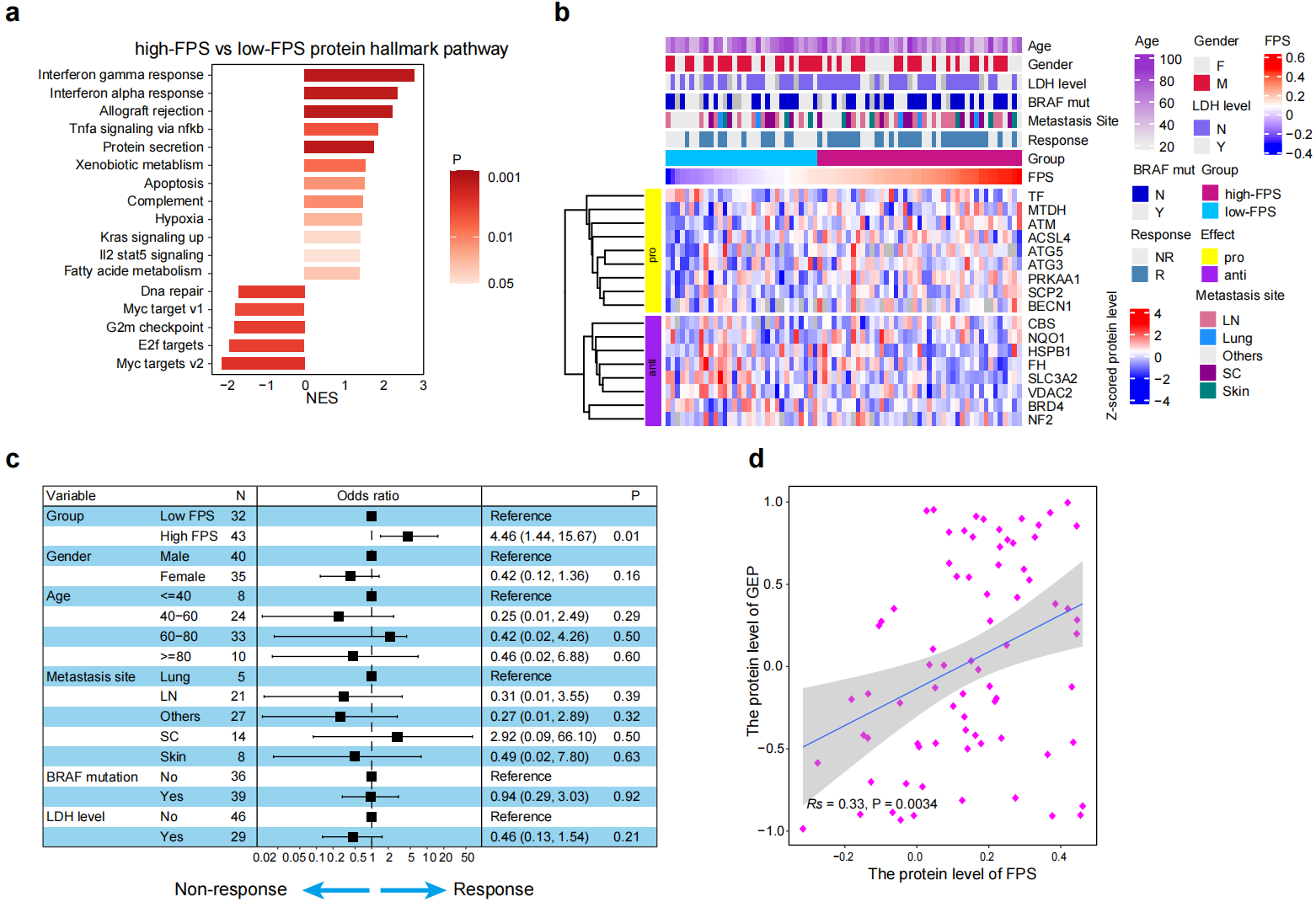


**Supplementary Figure 10. FPS was identified as a potential efficacy prediction biomarker for ICB treatment at the protein** **atlas.** (a) Enrichment of Cancer Hallmark (a) gene sets calculated by GSEA for protein expression. The color indicates the statistically significant enriched gene set (adjust P < 0.05). (b) The relative expression level of FRGs and the corresponding clinical features of individual patients. Heatmap annotation (upper) shows Clinical parameters of 75 PD1 treatment patients with advanced-stage melanoma, age, gender, LDH levels, *BRAF* mutation status, metastatic location, treatment response, FPS group and FPS are indicated. Heatmap shows the expression level of pro-FRGs and anti-FRGs that discriminate between R and NR group. Abbreviations are as follows: R: Response, including complete response (CR) and partial response (PR); NR: Non-Response, including stable disease (SD) and progressive disease (PD). (c) Multivariate logistic model analysis for response patients compared with non-response patients, which included the factors of FPS group, patient age, gender, LDH levels, *BRAF* mutation, and metastasis site. The length of the horizontal line represents the 95% confidence interval (CI) for each group. Squares represent odds ratio. The vertical dotted line represents the odds ratio of all patients shown by the forest plot. FRGs, ferroptosis associated genes; LDH, lactate dehydrogenase; pro-FRGs, pro-Ferroptosis genes; anti-FRGs, anti-Ferroptosis genes. (d) Scatter plot shows the spearman correlation between the FPS and GEP.


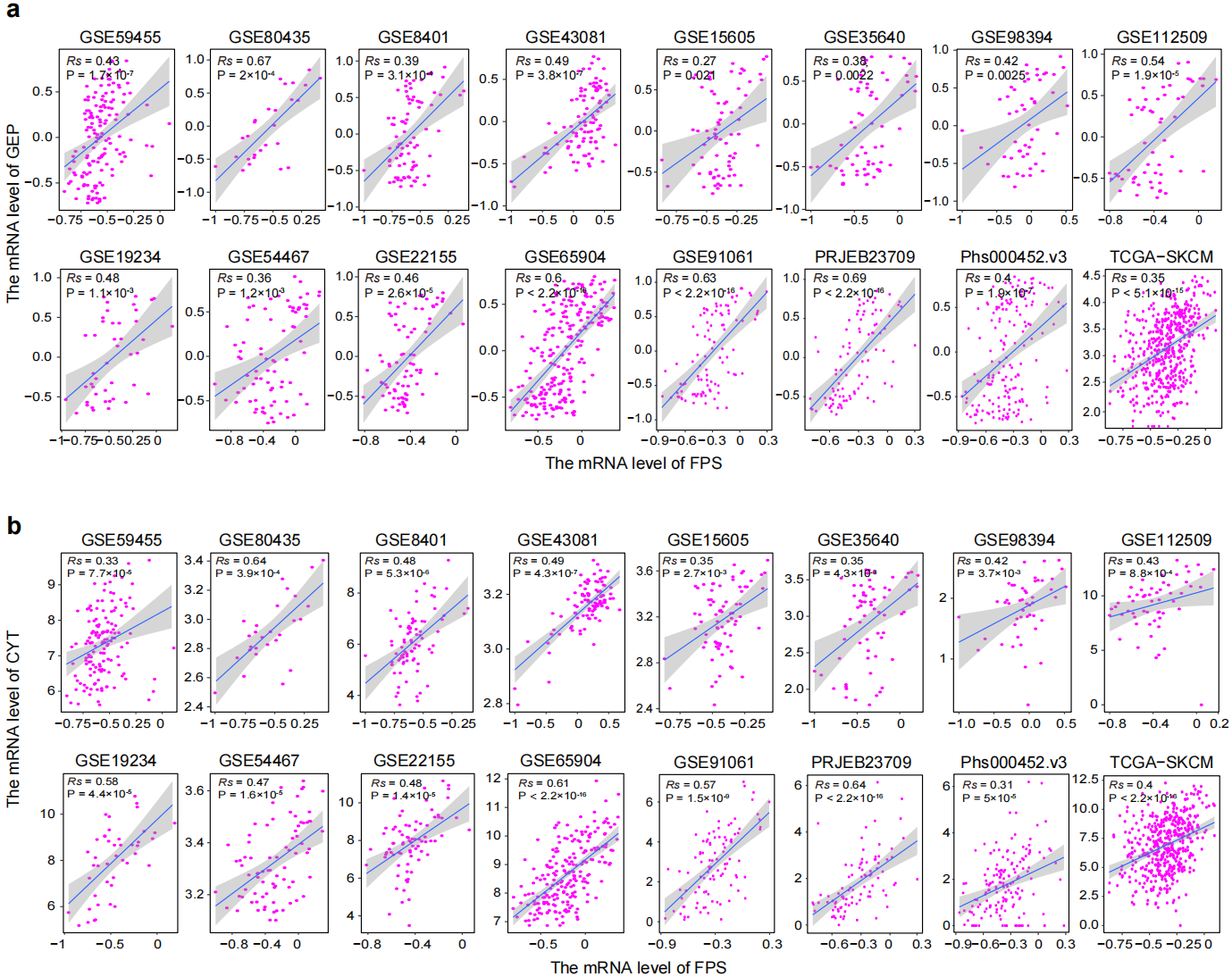
**Supplementary Figure 11. The association between FPS and GEP.** (a) Scatter plot shows the spearman correlation between the FPS and GEP. (b) Scatter plot shows the spearman correlation between the FPS and CYT.

**Supplementary Reference**

1. Kim, D., Paggi, J. M., Park, C., Bennett, C. & Salzberg, S. L. Graph-based genome alignment and genotyping with HISAT2 and HISAT-genotype. *Nat. Biotechnol.* **37**, 907–915 (2019).

2. Chang, K. *et al.* The Cancer Genome Atlas Pan-Cancer analysis project. *Nat. Genet.* **45**, 1113–1120 (2013).

3. Xiang, Y., Ye, Y., Zhang, Z. & Han, L. Maximizing the Utility of Cancer Transcriptomic Data. *Trends in cancer* **4**, 823–837 (2018).

4. Li, B. *et al.* Comprehensive analyses of tumor immunity: implications for cancer immunotherapy. *Genome Biol.* **17**, 174 (2016).

5. Zheng, X., Zhang, N., Wu, H.-J. & Wu, H. Estimating and accounting for tumor purity in the analysis of DNA methylation data from cancer studies. *Genome Biol.* **18**, 17 (2017).

6. Thorsson, V. *et al.* The Immune Landscape of Cancer. *Immunity* **48**, 812-830.e14 (2018).

7. Riaz, N. *et al.* Tumor and Microenvironment Evolution during Immunotherapy with Nivolumab. *Cell* **171**, 934-949.e16 (2017).

8. Jung, H. *et al.* DNA methylation loss promotes immune evasion of tumours with high mutation and copy number load. *Nat. Commun.* **10**, 4278 (2019).

9. Gide, T. N. *et al.* Distinct Immune Cell Populations Define Response to Anti-PD-1 Monotherapy and Anti-PD-1/Anti-CTLA-4 Combined Therapy. *Cancer Cell* **35**, 238-255.e6 (2019).

10. Liu, D. *et al.* Integrative molecular and clinical modeling of clinical outcomes to PD1 blockade in patients with metastatic melanoma. *Nat. Med.* **25**, 1916–1927 (2019).

11. Van Allen, E. M. *et al.* Genomic correlates of response to CTLA-4 blockade in metastatic melanoma. *Science* **350**, 207–211 (2015).

12. Beck, L. *et al.* Clinical Proteomics of Metastatic Melanoma Reveals Profiles of Organ Specificity and Treatment Resistance. (2021).

13. Tirosh, I. *et al.* Dissecting the multicellular ecosystem of metastatic melanoma by single-cell RNA-seq. *Science* **352**, 189–196 (2016).

14. Jerby-Arnon, L. *et al.* A Cancer Cell Program Promotes T Cell Exclusion and Resistance to Checkpoint Blockade. *Cell* **175**, 984-997.e24 (2018).

15. Sade-Feldman, M. *et al.* Defining T Cell States Associated with Response to Checkpoint Immunotherapy in Melanoma. *Cell* **175**, 998-1013.e20 (2018).

16. Zhao, E. *et al.* Spatial transcriptomics at subspot resolution with BayesSpace. *Nat. Biotechnol.* (2021).

17. Zhou, N. & Bao, J. FerrDb: a manually curated resource for regulators and markers of ferroptosis and ferroptosis-disease associations. *Database* **2020**, (2020).

18. Gaujoux, R. & Seoighe, C. A flexible R package for nonnegative matrix factorization. *BMC Bioinformatics* **11**, 367 (2010).

19. Hänzelmann, S., Castelo, R. & Guinney, J. GSVA: gene set variation analysis for microarray and RNA-Seq data. *BMC Bioinformatics* **14**, 7 (2013).

20. Basu, A. *et al.* An interactive resource to identify cancer genetic and lineage dependencies targeted by small molecules. *Cell* **154**, 1151–1161 (2013).

21. Seashore-Ludlow, B. *et al.* Harnessing Connectivity in a Large-Scale Small-Molecule Sensitivity Dataset. *Cancer Discov.* **5**, 1210–1223 (2015).

22. Yuan, Y. *et al.* Comprehensive Characterization of Molecular Differences in Cancer between Male and Female Patients. *Cancer Cell* **29**, 711–722 (2016).

23. Subramanian, A. *et al.* Gene set enrichment analysis: A knowledge-based approach for interpreting genome-wide expression profiles. *Proc. Natl. Acad. Sci.* **102**, 15545 LP – 15550 (2005).

24. Sergushichev, A. A. An algorithm for fast preranked gene set enrichment analysis using cumulative statistic calculation. *bioRxiv* 60012 (2016).

25. Yu, G., Wang, L.-G., Han, Y. & He, Q.-Y. clusterProfiler: an R package for comparing biological themes among gene clusters. *OMICS* **16**, 284–287 (2012).

26. Ayers, M. *et al.* IFN-γ-related mRNA profile predicts clinical response to PD-1 blockade. *J. Clin. Invest.* **127**, 2930–2940 (2017).

27. Roh, W. *et al.* Integrated molecular analysis of tumor biopsies on sequential CTLA-4 and PD-1 blockade reveals markers of response and resistance. *Sci. Transl. Med.* **9**, (2017).

28. Stuart, T. *et al.* Comprehensive Integration of Single-Cell Data. *Cell* **177**, 1888-1902.e21 (2019).
